# Coaching-Based Leadership Intervention Program: A Controlled Trial Study

**DOI:** 10.3389/fpsyg.2019.03066

**Published:** 2020-01-30

**Authors:** María Josefina Peláez Zuberbuhler, Marisa Salanova, Isabel M. Martínez

**Affiliations:** WANT Research Team, Universitat Jaume I, Castelló de La Plana, Spain

**Keywords:** coaching leadership, psychological capital, work engagement, performance, control trial

## Abstract

In spite of the potential benefits that coaching-based leadership interventions can bring to organizations, basic questions remain about their impact on developing coaching skills and increasing psychological capital (PsyCap), work engagement and in- and extra-role performance. In a controlled trial study, 41 executives and middle managers (25 in the experimental group and 16 in the waiting-list control group) from an automotive sector company in Spain received pre-assessment feedback, a coaching-based leadership group workshop, and three individual executive coaching sessions over a period of 3 months. The intervention program used a strengths-based approach and the RE-GROW model, and it was conducted by executive coaching psychologists external to the organization. Participants (*N* = 41) and their supervisors (*N* = 41) and employees (*N* = 180) took part in a pre-post-follow up 360-degree assessment during the research period. Quantitative data were analyzed using Analyses of Variance (ANOVA) with a 2 × 2 design, paired-samples *t*-tests, and univariate analyses between groups. Results indicated that the intervention program was successful in increasing the participants’ coaching-based leadership skills, PsyCap, work engagement, and in- and extra-role performance. Qualitative measures were also applied, and results from individual responses provided additional support for the study hypotheses. Regarding practical implications, the results suggest that the Coaching-based Leadership Intervention Program can be valuable as an applied positive intervention to help leaders develop coaching skills and enhance well-being and optimal functioning in organizations.

## Introduction

The rapid changes and advances in economic, political, technological, and social factors (Kirchner and Akdere, 2014) require managers in organizations to develop human capital in order to achieve strategic organizational goals (Kim, 2014). This complex and challenging context also creates the need to develop healthy and positive leaders who are able to maintain and optimize psychosocial wellbeing in organizations (Salanova et al., 2012).

Moreover, research increasingly shows that being an effective leader means being an effective coach (Goleman et al., 2012; Grant and Hartley, 2014). Thus, good coaching skills are becoming an essential part of effective leadership and positive workplace cultures (Ellinger et al., 2011; Stehlik et al., 2014). In such cultures, coaching is the main style of managing and working with others, with a predominant commitment to employees’ growth (Underhill et al., 2007; Wood and Gordon, 2009). Currently, organizations are starting to invest in training to develop coaching skills in their managers and leaders (Milner et al., 2018) in order to enhance wellbeing and performance and facilitate organizational and personal change (Ellinger et al., 2003; Wright, 2005; Grant and Cavanagh, 2007a).

Previous studies have highlighted *coaching-based leadership* (also known as leader-as-coach or managerial coaching) as a key indicator of effective managerial behavior to influence employees without relying on formal authority (Hamlin et al., 2006; Ellinger et al., 2008; Pousa et al., 2018). Specifically, leaders as coaches have been identified as crucial in developing and empowering employees due to the high cost of external coaching and the need to become learning organizations and innovate to stay competitive (Segers et al., 2011; Kim, 2014). For these reasons, organizations are transferring responsibilities of Human Resources Development practitioners, such as coaching, to their leaders (Liu and Batt, 2010; Kim, 2014). In this study, the term coaching-based leadership will be used to refer to the leader, manager, or supervisor in their roles as coaches or when using coaching skills in work settings.

Despite the growing popularity of coaching-based leadership interventions (Milner et al., 2018), the efficacy of these programs and their impact on the development of effective leaders have rarely been assessed (Ellinger et al., 2011; Grant and Hartley, 2014; Berg and Karlsen, 2016). Indeed, previous research has revealed that only one-third of these initiatives are evaluated (Ely et al., 2010). Although there are good initiatives and significant investments in leadership skill development programs, organizations still believe they have not effectively trained their leaders. In fact, they continue to report a lack of leadership skills among their employees (Lacerenza et al., 2017). Research has shown that leaders need at least 3 to 6 months to develop coaching skills and feel comfortable using them (Grant, 2010). So far, very little is known about the benefits of developing a coaching-based leadership style and its impact on work-related outcomes (Berg and Karlsen, 2016) such as psychological capital (PsyCap), work engagement and in-role and extra-role performance.

Moreover, effective methodologies for teaching and training coaching skills in organizations have to be further developed (Ellinger et al., 2003; Segers et al., 2011). There is also a need for empirical studies with strong designs and mixed methodologies (qualitative and quantitative) to investigate possible effects of these intervention programs over time (Grant and Hartley, 2013, 2014). Previous research has highlighted the value of qualitative approaches in the evaluation of the human process of coaching because they can lead to the discovery of novel themes and new insights about a topic under investigation (Coe, 2004; Gyllensten and Palmer, 2007). To address this research gap, we conducted a controlled trial Coaching-based Leadership Intervention Program and explored its impact on leaders’ coaching skills, PsyCap, work engagement, and in- and extra-role performance over time, using a 360-degree assessment.

## Theory and Hypotheses

### Defining Coaching-Based Leadership

Coaching can be understood as a collaborative relationship between coach and coachee, oriented toward facilitating goal attainment and individual change (Spence and Grant, 2007). In the specific work context, coaching is generally provided by the leader as a way to enhance employees’ goal achievement and performance through the use of a variety of emotional, cognitive, and behavioral techniques (Grant, 2010). Grounded theoretically in coaching leadership theory, this recently form of leadership has been defined as a day-to-day process of providing support, and helping employees identify opportunities to achieve individual development goals (Cox et al., 2010; Berg and Karlsen, 2016). Leaders who succeed with a coaching style enable employees to gain awareness and reflection, generate their own answers (Cox et al., 2010; Milner et al., 2018), require less control and directing, and have a desire to help them develop and flourish (Berg and Karlsen, 2016). Goleman et al. (2012) suggested that coaching is one of the leadership styles that achieves the best results, and that its main purpose is to develop employees’ personal resources. Coaching leaders are oriented toward helping employees strengthen their talents by paying attention to their needs and building an effective alliance (Dello Russo et al., 2017). From a psychosocial perspective, coaching provided by leaders is suggested as an important job (social) resource that facilitate a motivational process that enhances the development of personal resources, leading to work engagement and better performance (Schaufeli and Bakker, 2004).

As noted by Ellinger et al. (2005), the coaching leadership style offers organizations a theoretical foundation for adopting a people-oriented approach in the relationship with employees. This recent theory on leadership has been developing away from other leadership approaches, such as transactional or transformational, toward a new paradigm that seeks to reduce the differentiation between the leader and the employee (Hagen and Aguilar, 2012). For instance, Bass and Avolio’s (1994) transformational leadership style is essentially about motivating followers to look beyond their own self-interest toward the achievement of team-related goals (Bormann and Rowold, 2018). In contrast, leaders’ coaching behaviors refer to one-on-one interactions between a leader and an employee aimed at stimulating individual growth (Anderson, 2013) and may therefore be more suitable for addressing personal and professional developmental goals (Kunst et al., 2018).

Given the little guidance that coaching-based leaders receive in their own growth and development, along with the limited number of frameworks to support this process, Kemp (2009) emphasized the need for leaders as coaches to be guided by a personal understanding of their expected responses in order to enhance change. This author proposed a coaching and leadership alliance framework to contextualize the coaching leadership process and clarify its role in helping employees to strengthen their potential. According to this theoretical proposal, leaders engage in a process similar to that of coaches by engaging in an alliance-building process with employees that leads to a deep sense of shared meaning. As a result of this alliance, the coaching leader facilitates work-related outcomes and fosters new ways to achieve performance.

The coaching leader displays a set of skills or beliefs that can support a coaching mentality that enables the execution of specific actions or behaviors toward their employees (David and Matu, 2013). In order to enhance optimal functioning, organizations increasingly ask their managers and leaders to develop specific skills such as effective communication, empathy, or trust, promote goal achievement, and enhance professional and personal change (Ellinger and Bostrom, 2002; Mai and Akerson, 2003; Grant, 2010; Grant and Hartley, 2013; Berg and Karlsen, 2016). According to the International Coach Federation (n.d.), the leading global coaching organization, essential coaching competencies consist of establishing trust and a working alliance, active listening, powerful questioning, direct communication, designing actions and goal setting, and managing progress. In using coaching skills, leaders enable employees to generate their own answers, thus enhancing development and performance (Grant and O’Connor, 2010; Milner et al., 2018). In the current study, we follow previous literature and research related to the professional coach’s skills, the leader- as- coach, and managerial coaching, in order to identify eight core coaching-based leadership skills classified into four dimensions: (a) *working alliance*: developing a working alliance (1); (b) *open communication*: active, empathic, and compassionate listening (2), and powerful questioning (3); (c) *learning and development*: facilitating development (4), providing feedback (5), and strengths spotting and development (6); and (d) *progress and results*: planning and goal setting (7), and managing progress (8).

#### Working Alliance

Developing a working alliance refers to the ability to create a safe environment that contributes to the establishment of mutual respect, sincerity, trust, and transparency (Graham et al., 1994; Gyllensten and Palmer, 2007). Previous coaching and managerial coaching literature has highlighted the essential role of trust in the coaching relationship (Hunt and Weintraub, 2002; Ting and Riddle, 2006; Gregory and Levy, 2011). Effective coaching involves showing genuine interest in employees’ wellbeing and future, continually demonstrating sincerity, establishing clear agreements, and keeping promises. This skill is essential for leaders because it allows them to develop partnerships and build warm, friendly relationships with employees (Graham et al., 1994). As a result, shared meaning, purpose and commitment emerges, allowing for high levels of mutual engagement to drive opportunities and achieve performance (Kemp, 2009).

#### Open Communication

*Open communication* is considered one of the key factors leading to effective coaching (Park et al., 2008). This dimension refers to the use of effective communication techniques to establish a good rapport with employees and facilitate personal and professional potential and performance (Gilley et al., 2010). Specifically, leaders as coaches engage in formal or informal conversations using techniques such as asking powerful questions, and active, empathic, and compassionate listening (Whitmore, 1992; Graham et al., 1994; Gilley et al., 2010). Question framing is considered an essential coaching-based leadership behavior that encourages employees to think through issues (Ellinger et al., 2003). Adequate questions are those that stimulate motivation and subsequently elicit deeper awareness and reflection (Kemp, 2009). Likewise, appropriate levels of empathy, understanding, compassion, and acceptance create an environment where employees can feel free to express their emotions and ideas (Graham et al., 1994; Grant and Cavanagh, 2007a; Kemp, 2009). With the leader’s help, employees gain awareness, engage in reflection, and increase their ability to take responsibility for their own development (Gilley et al., 2010).

#### Learning and Development

Facilitating development refers to the ability to provide support and training to employees in order to encourage their progress and continuous learning and effectively lead them toward the desired results (Park et al., 2008; Berg and Karlsen, 2016). As Ellinger and Bostrom (2002) observed, a predominant behavior in coaching-based leadership involves creating and promoting a learning environment, for instance, by providing feedback and helping employees to identify, build and use personal strengths (Berg and Karlsen, 2016). In doing so, they encourage employees to better direct their talents and abilities toward meaningful and engaging behaviors (Peterson and Seligman, 2004). In essence, employees who use their strengths are more engaged at work (Harter et al., 2002) and more likely to achieve their goals (Linley et al., 2010b).

#### Progress and Results

Planning and goal setting refers to the ability to support employees in establishing individual development goals that are valued by them, and ensure that they complete the agreed-upon action steps (Grant and Cavanagh, 2007b). Previous research has indicated that leaders as coaches work collaboratively with each employee to set engaging, challenging goals that motivate performance (Dahling et al., 2016). Finally, managing progress requires leaders to monitor, re-define, and evaluate employee action plans and performance, and manage both responsibilities in the process (Grant, 2003; Grant and Cavanagh, 2007b).

### Coaching-Based Leadership Intervention and Its Efficacy

In their meta-analysis on the impact of leadership, Avolio et al. (2009) defined leadership interventions as focusing on manipulating leadership as the independent variable through training, assignments, or other means. The authors indicated that the most common aim of these interventions is leadership training and development. Further research has suggested that leadership intervention programs should focus on knowledge and skills that can enhance leader effectiveness (Amagoh, 2009). These interventions have generally involved training in a workshop format, participation in executive coaching, or a combination of these two approaches (Kelloway and Barling, 2010; Lacerenza et al., 2017).

There has been some question about how leaders can be led to display a coaching-based leadership style. Specifically, leader-as-coach training programs aim to enhance leadership quality in organizations by providing training in coaching skills (Graham et al., 1994; Hagen, 2012; Grant and Hartley, 2014). The increased demand for leaders with coaching skills is generally attributed to the many recognized benefits, such as enhanced employee and organizational performance (Liu and Batt, 2010; Ellinger et al., 2011; Kim et al., 2013; Tanskanen et al., 2019). Additionally, previous studies have identified leaders as coaches as a powerful developmental intervention for motivating, developing, and retaining employees in organizations (Ellinger et al., 2011). Although leaders are often expected to apply coaching principles at work, and many of them express a desire for further training, these developmental programs do not always focus on specific coaching skills. In fact, to be operational, training needs to align these skills with personal and professional goals (Milner et al., 2018).

The second approach involved in leadership interventions, executive coaching, is an increasingly popular approach to help executives develop leadership skills or behaviors and improve their performance and, therefore, the performance of the organization as a whole (Feldman and Lankau, 2005; Gray, 2006). The number of organizations using executive coaching to develop leaders increases every year because it is considered one of the dominant methodologies for developing effective leaders (Grant, 2013). An effective way to support leadership development in organizations is the strengths-based leadership coaching approach (MacKie, 2014). This approach is based on positive psychology discipline, which focuses on developing positive qualities, rather than dealing with negative aspects such as weaknesses and pathologies (Seligman and Csikszentmihalyi, 2014). Strengths-based coaching is based on the identification, development, and use of personal strengths in order to foster positive outcomes such as goal attainment, optimal functioning, fulfillment, and well-being (Linley et al., 2010b). Specifically in leadership development, this approach provides a structure that includes strength awareness and balance, pairing strengths with leadership skills, and aligning them with personal or organizational goals (MacKie, 2014).

The use of coaching behaviors as a performance enhancement method has gained popularity in organizations (Boyatzis et al., 2013; Dimas et al., 2016). However, relatively few empirical studies have attempted to examine the efficacy of training and developing leaders as coaches (Grant, 2006). This is surprising because previous researchers reported that leadership interventions could be useful in developing and improving coaching skills (Styhre, 2008; Ellinger et al., 2010). In one of these studies, David and Matu (2013) found a positive impact of a managerial coaching program on increasing coaching abilities reported by the managers themselves and by external observers. Similarly, in the Cummings et al. (2014) quasi-experimental study, leaders’ attitudes and intentions to be a coach increased significantly after participating in a workshop on how to coach their employees.

Although there has been an increase in the number of studies on this topic, there continues to be a call for more empirical investigation on the way leaders are being trained in coaching skills (Milner et al., 2018) and on the effectiveness of these intervention programs. Additionally, there is still a need to develop effective methodologies for training and assessing these interventions (Cavanagh and Grant, 2004; Grant and Hartley, 2013, 2014; Day et al., 2014). To fill this gap, in a controlled trial study, we tested the effects of a Coaching-based Leadership Intervention Program on essential coaching skills. A 360-degree format evaluation was applied that includes self-assessment along with employees’ and supervisors’ evaluations of the leader’s coaching skills. Considering different insights is important in order to have diverse views of the training outcomes and efficacy (Milner et al., 2018).

Hypothesis 1 (H1): Participants’ levels of coaching-based leadership skills will increase after the intervention (POST) compared to their baseline levels (PRE) and compared to the waiting-list control group (WL).

### Coaching-Based Leadership and PsyCap

According to the Conservation of Resources theory (Hobfoll, 2002), individuals seek to obtain, retain, and protect personal resources to control and impact upon their environment successfully. Based on this theory, Luthans et al. (2007, 2015) refer to PsyCap as a positive personal resource and defined it as an individual’s positive psychological state of development that is comprised of: (1) self-efficacy; having confidence to mobilize the motivation, cognitive resources or courses of action needed to successfully executive challenging tasks; (2) hope: persevering toward goals, and identifying alternative ways to reach goals in order to succeed; (3) resilience: the capacity to bounce back from adversity to attain success; and (4) optimism: making a positive attribution about succeeding in the present and in the future (Luthans et al., 2015). Although these four psychological resources are conceptually distinct, they combined into a higher-order construct in which they interact in a synergetic way. As a result of the investment of such set of psychological resources, individuals obtain experiential rewards from the present moment while also increasing the likelihood of future benefit (Kersting, 2003).

Based on the Job Demands-Resources (JD-R) model, Bakker and Demerouti (2007) claimed that job resources, such as supervisory coaching and opportunities for professional development, play an intrinsic motivational role fostering employees’ growth, learning and development, thus suggesting that such job resources foster the development of personal resources. In line with this proposition, Goleman et al. (2012) argued that the main purpose of coaching leaders is to develop employee’s personal resources. Leaders do so in daily interactions by paying attention to their employees needs, developing a trust environment, building an effective alliance, and providing personalized learning (Ellinger et al., 2011; Dello Russo et al., 2017). In other words, leaders can foster PsyCap through the use of specific coaching skills. Previous research has shown a positive direct link between job resources such as coaching provided by leaders and specific personal resources (i.e., self-efficacy, organizational-based self-esteem and optimism; Xanthopoulou et al., 2007). A recent study has examined and confirmed the positive direct relationship between managerial coaching and employees’ PsyCap (Hsu et al., 2019). Furthermore, Pitichat et al. (2018) highlighted the significant relationship between the leaders self-development and their levels of PsyCap, thus resulting in enhanced chances of success at work. However, there is still a lack of studies that empirically examined the impact of a coaching-based leadership intervention on the leaders’ PsyCap. This is important because there is growing evidence that PsyCap plays an important role in improving positive work attitudes and behaviors (Luthans et al., 2010).

Hypothesis 2 (H2): Participants’ levels of PsyCap will increase after the intervention (POST), compared to PRE and compared to the WL.

### Coaching-Based Leadership and Work Engagement

Research on leadership and coaching that analyses the relationship between coaching skills and well-being related outcomes, such as employees’ job satisfaction, is on the rise (Ellinger et al., 2003, 2011; Kim et al., 2013). However, fewer studies have attempted to explore the impact of coaching-based leadership skill training and development on engagement in the work field. Work engagement is defined as a positive, fulfilling, work-related state of mind characterized by three dimensions: (1) vigor: which refers to high levels of energy and mental resilience while working, the willingness to invest effort in one’s work, and persistence when facing difficulties; (2) dedication: which refers to strong involvement and psychological identification with one’s work, characterized by a sense of significance, enthusiasm, pride, inspiration, and challenges; and (3) absorption: which refers to a state of full concentration and being engrossed in one’s activities, where time passes quickly and it becomes difficult to separate oneself from work (Schaufeli et al., 2006). Based on the JD-R model (Bakker and Demerouti, 2007), work engagement arises from a motivational process that begins with the availability of job and personal resources that stimulate employees’ motivation and, therefore, leads to desirable work outcomes such as organizational commitment and higher job performance (Llorens-Gumbau and Salanova-Soria, 2014).

Practitioner literature has highlighted the potential of leadership behavior as a key driver in enhancing engagement (Shuck and Herd, 2012). In line with MacLeod and Clarke’s (2009) research, leaders promote engagement by providing employees with autonomy, empowerment, and developmental opportunities, offering them coaching and feedback, and ensuring that the work is effectively and efficiently designed. When the leader provides coaching, employees are more engaged with their work because they receive more guidance from their leader in achieving their goals (Kim, 2014). Although there are few studies on this link, research exploring the association between coaching-based leadership and employee work engagement is increasing. For instance, Ladyshewsky and Taplin (2017, 2018) found a significant positive relationship between these constructs. Further studies demonstrated a mediating role of work engagement in the relationship between the leader’s coaching and performance-related outcomes (Lin et al., 2016; Ali et al., 2018; Lee et al., 2018; Tanskanen et al., 2019). Despite interesting findings, all these studies are cross-sectional, and work engagement is evaluated as an employee-related outcome.

With only one exception (Grant and Hartley, 2014), research exploring the impact of leader-as-coach development programs on increasing the leaders own work engagement is still missing. This is surprising because engagement is generally associated with core aspects of coaching, such as generating meaningful and positive feedback, goal clarity, and effective leader-employee communication (Bakker et al., 2008; Grant and Hartley, 2014). Moreover, previous research has highlighted the positive impact of training on individuals’ self-efficacy (Holladay and Quiñones, 2003), which in turn generates the perception of challenging demands, positive job resources, and higher levels of engagement with work (Ventura et al., 2015). Accordingly, when leaders have high levels of energy, vitality, and engagement, they are likely to invest more effort in their activities and tasks and, therefore, in practicing their leadership skills at work (Kark, 2011). Thus, focusing on the leader’s work engagement, we hypothesize the following:

Hypothesis 3 (H3): Participants’ levels of work engagement will increase after the intervention (POST), compared to PRE and compared to the WL.

### Coaching-Based Leadership and In-Role and Extra-Role Performance

Job performance generally includes two dimensions: in-role or task performance and extra-role or contextual performance. Although other types of job performance may be integrated into the concept, such as adaptive, innovate, or service performance, in this study we focus on in-role and extra-role performance which are considered as key healthy organizational outcomes from a psychosocial perspective (Salanova et al., 2012). In-role performance refers to activities that are related to the formal job and directly serve the goals of the organization (Goodman and Svyantek, 1999). According to the JD-R model, the extrinsic motivational potential of job resources, such as supervisor support, fosters employees to meet their goals, and become more committed to their job because they derive fulfillment from it (Bakker and Demerouti, 2007). Previous research has specified the role of managerial coaching in improving employee in-role performance by clarifying goals and providing resources to achieve them (Kim, 2014; Kim and Kuo, 2015). Leaders who act as role models, deliver instant feedback, and assist employees in the learning processes help to improve employees’ task performance. Related to this assumption, previous research revealed a positive and direct link between supervisory coaching skills and employee in-role performance (Ellinger et al., 2003, 2005, 2011; Agarwal et al., 2009; Liu and Batt, 2010). Further studies also found an indirect effect of managerial coaching on task performance (Kim et al., 2013; Kim and Kuo, 2015).

Whereas in-role performance describes technical core behaviors, extra-role performance denotes actions that exceed what the employee is supposed to do, such as helping others or voluntary overtime (Goodman and Svyantek, 1999). This contextual-related performance refers in part to citizenship behaviors that directly promote the effective functioning of an organization without necessarily directly influencing an employee’s productivity (Podsakoff et al., 2000). Specific leader coaching skills, such as open communication with employees (Podsakoff et al., 2000; Bester et al., 2015) and one-on-one interactions, encourage employees to perform extra-role behaviors in the organization (Raza et al., 2017). From a social exchange perspective, the leader- as- coach is considered a form of organizational support (Kim, 2014; Kim and Kuo, 2015) that positively influences organizational citizenship behaviors (Ellinger and Cseh, 2007; Kim and Kuo, 2015).

Previous research has indicated that training to enhance the knowledge, skills, and abilities of individuals leads to an increase in performance in the work setting (Holladay and Quiñones, 2003). Although coaching can be perceived as time-consuming, the development of effective workplace coaching skills leads to increased performance at both managerial and supervisory levels (Graham et al., 1994; Grant, 2010). However, only a few studies have examined the impact of leader coaching skill interventions on job performance (Cummings et al., 2014; Grant and Hartley, 2014; Ratiu et al., 2017). Indeed, recent research has focused more on the effects of coaching-based leadership interventions on employees’ performance, rather than examining the impact on the leader’s own performance (Grant, 2010). Moreover, the few studies that have examined the impact of leader- as-coach interventions (Moen and Skaalvik, 2009; David and Matu, 2013; Grant and Hartley, 2014; Ratiu et al., 2017) have considered performance as a whole, without distinguishing between task and contextual dimensions. In the current study, we focus on leaders’ in-role and extra-role performance as perceived by their supervisors and employees.

Hypothesis 4a (H4a): Participants’ levels of in-role performance will increase after the intervention (POST), compared to PRE and compared to the WL.

Hypothesis 4b (H4b): Participants’ levels of extra-role performance will increase after the intervention (POST), compared to PRE and compared to the WL.

### The Durability of the Effects

In order to truly assess the effectiveness of an intervention, it is necessary to evaluate whether or not the reported effects are maintained over time (Grant and Hartley, 2013). Despite the significant investment in training programs in leadership skills, organizations continue to report a lack of leadership skills among their employees in the workplace (Lacerenza et al., 2017). Because leaders need time to develop and apply coaching skills in the workplace (Grant, 2010; Grant and Hartley, 2013), it is always a challenging task for facilitators and practitioners to ensure that the skills developed during training are actually transferred to the workplace (Burke and Baldwin, 1999; Grant and Hartley, 2013). Therefore, previous researchers have highlighted the need to explore the long-term impact of leader-as-coach interventions (Kirchner and Akdere, 2014; Milner et al., 2018). Only a few scholars have demonstrated a long-term sustained influence of a leader-as-coach program on improvements in coaching skills and engagement (Grant and Hartley, 2014).

Not surprisingly, the development of effective methodologies for providing training in coaching-based leadership skills can facilitate positive organizational change, leading to higher levels of productivity and engaging workplace environments (Grant and Hartley, 2013). The majority of the quasi-experimental studies carried out to date have examined the effects of these interventions on performance-related outcomes immediately after participation (Moen and Skaalvik, 2009; Ratiu et al., 2017). However, none of these studies evaluated the long-term sustained impact after a certain number of months had passed (follow up) since the intervention. Thus, in the current study, we attempt to investigate the durability of the intervention program’s effects on the outcome variables (coaching-based leadership skills, PsyCap, work engagement, and in- and extra-role performance) over time (FUP; Follow Up time; 4 months after finishing the program).

Hypothesis 5: Participants’ levels of coaching-based leadership skills (H5a), PsyCap (H5b), work engagement (H5c), and in- and extra-role performance (H5d), will remain higher at FUP, compared to PRE intervention.

## Materials and Methods

### Participants and Procedure

The study was conducted in a multinational automotive industry company in Spain. The plant had 42 managers and middle managers, all of whom were invited to participate in the program through informational meetings held by university researchers. During these meetings, participants were informed about the nature of the study and the aims of the intervention. There were no additional economic rewards or employee benefits in exchange for their involvement in the study. They were asked to take part voluntarily, with the confidentiality of their replies guaranteed, and 41 of them (97%; 15 managers and 26 middle managers) initially agreed to participate. The study adhered to ethical standards and was approved by the University Research Ethics Committee.

Next, participants were distributed into the *experimental group* (EX; *N* = 25) and the *waiting-list control group* (WL; *N* = 16). Two simultaneous workshop groups were assigned to the EX, one for the managers (*N* = 15) and the other for the middle managers (*N* = 10), with one person dropping out in each group after the first individual coaching session. The groups were not randomly chosen because the managers have management responsibilities that affect middle managers; therefore, the company decided to separate the two groups. The WL served as an untreated comparison group during the study. After the EX had ended, 15 members of this WL also participated in the intervention program, with only one person dropping out after the workshop ended. Thus, the final sample consisted of 37 participants (EX = 23; WL = 14). For organizational reasons, the WL started the intervention immediately after the EX finished it, rather than waiting until the FUP assessment took place.

**FIGURE 1:**
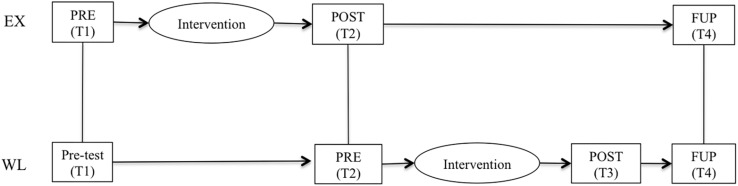
Experimental design of the study. EX experimental group; WL waiting list-control group; PRE pre-assessment; POST post-assessment; FUP follow up-assessment; Tl time 1; T2 time 2; T3 time 3; T4 time 4.

Participants (*N* = 41) and their supervisors (*N* = 41) and employees (*N* = 180) were asked to answer an online research questionnaire at different times (three times by the EX and four times by the WL) during the research period: (1) before starting the intervention, the EX (Time1: pre-assessment for the whole intervention group; participants: *N* = 41; supervisors: *N* = 38; employees: *N* = 180); (2) immediately after finishing the intervention, the EX, and before the WL started (Time 2: post-assessment for EX and pre-assessment for WL; participants: *N* = 40; supervisors: *N* = 38; employees: *N* = 117); (3) immediately after finishing the intervention, the WL (Time 3: post-assessment just for WL; participants: *N* = 14; supervisors: *N* = 14; employees: *N* = 53); and 4 months after finishing the intervention each group (Time 4: follow up assessment for the whole intervention group; participants: *N* = 37; supervisors: *N* = 33; employees: *N* = 90). All the study variables (coaching-based leadership skills, PsyCap, work engagement, in- and extra-role performance) were assessed at the four different times. Figure 1 outlines the research design of the study.

The participants’ coaching-based leadership skills were both self- reported and evaluated by their supervisors and employees, in a 360-degree format. Additionally, only participants assessed their levels of work engagement. Furthermore, supervisors’ and employees’ ratings of the participants’ performance were included in order to obtain an external performance assessment and avoid common method bias. Finally, during the last individual sessions, qualitative data were gathered through open questions.

Regarding the demographic breakdown of the subjects, 88% were men, with a mean age of 45 years (SD = 9.3, ranging from 28 to 63). Moreover, 100% had a tenured contract, and the average tenure in the company was 16.5 years (SD = 10.8).

### Coaching-Based Leadership Intervention Program Description

Participants took part in a “Coaching-based Leadership Intervention Program” over a period of 3 months. The main goal of the program was to support the development and improvement of the managers’ and middle managers’ coaching skills. The intervention was delivered in a group workshop format, followed by three individual executive coaching sessions.

The group workshop consisted of five 180-min weekly group sessions. In the first session, feedback about the PRE-assessment questionnaire results (coaching-based leadership skills, PsyCap, work engagement, and in- and extra-role performance variables) was given. Next, participants received academic input related to positive organizational psychology (Salanova et al., 2016) and emotional appraisal and regulation, given that every leader has to have the ability to manage his/her emotions and consider others’ emotions when directing actions (Goleman et al., 2012). Previous research considered emotional regulation to be an important factor influencing general leadership effectiveness (Gooty et al., 2010). Next, participants received emotional regulation practice based on role-playing activities and mindfulness techniques (Tan, 2012; Kashdan and Ciarrochi, 2013; Hanson, 2017). By receiving training in this generic leadership skill, participants were then prepared to receive training in specific coaching skills. In addition, a booklet was provided that contained work slogans, relevant information for each week’s instruction, and suggested reading materials.

The following four sessions combined academic input and practicing a coaching-based leadership skillset through role-playing among participants and with the use of the skills on-the-spot with their employees. Based on the pre-assessment results and the workshop contents, during session 2 participants established a goal related to the development or improvement of their coaching-based leadership skills. Additionally, they received theory and practice related to developing a working alliance (Gyllensten and Palmer, 2007; Acosta Antognoni et al., 2012) and open communication (Whitmore, 1992; Neff, 2003; Hoffman et al., 2008; Tan, 2012; Boyatzis et al., 2013; Gilbert, 2013) skills. During session 3, theory and practice related to facilitate development, providing feedback and strengths spotting and development skills was delivered (Park et al., 2008; Berg and Karlsen, 2016). During this session, participants worked on the identification, development and use of personal strengths, based on the VIA (Values In Action) inventory of strengths, the identification of strengths through answering open questions (e.g., “of what are you most proud?”) in pairs, and the establishment of a strengths in action plan to be developed at work (Peterson and Seligman, 2004; Biswas-Diener, 2010; Meyers and van Woerkom, 2017). During session 4, the participants received academic inputs and practice related to planning goals and managing progress skills (Grant, 2003; Grant and Cavanagh, 2007b). Based on the Goal, Reality, Option, Wrap-up (GROW) model (Grant, 2011), the participants explored options in order to achieve the goal set during session 2, and established an action plan to be reviewed during the individual coaching process. Finally, a brief 2-h closing session took place with the objective of savoring the positive experiences that occurred during the workshop. A future ‘best possible self’ (Peters et al., 2013) visualization exercise related to developing a coaching-based leadership style was delivered to strengthen the resulting improvements and foster the motivation to continue working on goal achievement during the coaching process. Participants also gave written qualitative feedback about their experiences in the workshop and the key learning points. The specific workshop contents and structure are presented in Table 1.

**TABLE 1:** Specific workshop session contents.

Workshop session no	Topics	Activities	Homework
1	Positive psychology and coaching-based leadership skills Workplace coaching Emotion appraisal and regulation as a generic leadership skill	Welcome: presentation, objectives, structure and internal rules of the program Pre-assessment results: feedback and reflection Role-playing and mindfulness practice Booklet provided with work-session slogans, the week’s instruction, and suggested reading materials	Self-compassion test (online) Field weekly to practice emotion appraisal and regulation
2	GROW Model: phase 1: Goal setting (SMART+goals) Skill no 1: Development of a working alliance Skill no 2: Active, empathic, and compassionate listening Skill no 3: Powerful questioning	Brief mindfulness practice Role-playing in pairs: setting goal related to the development and/or progress of coaching-based leadership skills Self-compassion test results and reflection Role-playing in pairs: practicing effective listening and questioning	VIA Inventory of Strengths (online) Field weekly to practice skill no 1 and skill no 2
3	Skill no 4: Facilitate development Skills no 5: Providing feedback Skill no 6: Strengths spotting and development GROW Model: phase 2: Examine Reality: Personal strengths, weaknesses, opportunities and threats (or limitations)	Brief mindfulness practice VIA inventory of strengths results and reflection Role-playing in pairs: detect and develop strengths Choice of key personal strengths. Strengths in action Role-playing: practicing structured feedback process	SWOT: analysis of Strengths, Weaknesses, Opportunities and Threats Field weekly to practice skill no 3
4	GROW Model: phase 3: Explore Options, and phase 4: Establish the Will. Skill no 7: Planning and goal setting Skill no 8: Managing progress	Table of alternatives: advantages and disadvantages Action plan: establish and develop an action plan for goal achievement	Field weekly to practice skill no 4
5	Closing, review, and reflection	Topics, booklet exercises and field weekly review Follow-up of the action plan Future BPS (Best Possible Self) exercise and visualization	Public image: ask co-workers and employees to complete files with strengths and improvement areas

After the workshop, the participants went through an executive micro-coaching process based on a previous validated strengths-based micro-coaching intervention (see Peláez et al., 2019), which consisted of three biweekly 90-min individual sessions with a professional coaching psychologist external to the organization. Previous research has confirmed that coaching can be effective even when the number of coaching sessions is relatively low (Theeboom et al., 2014; Peláez et al., 2019). The individual coaching sessions aim to support participants during the development of an action plan related to the goal they set during the workshop, related to the improvement of their coaching skills. The coaching process followed a strengths-based leadership coaching approach, based on the identification, development, and use of personal strengths (Govindji and Linley, 2007; Linley et al., 2010b) and alignment with leadership skills (MacKie, 2014) to foster positive outcomes.

**FIGURE 2:**
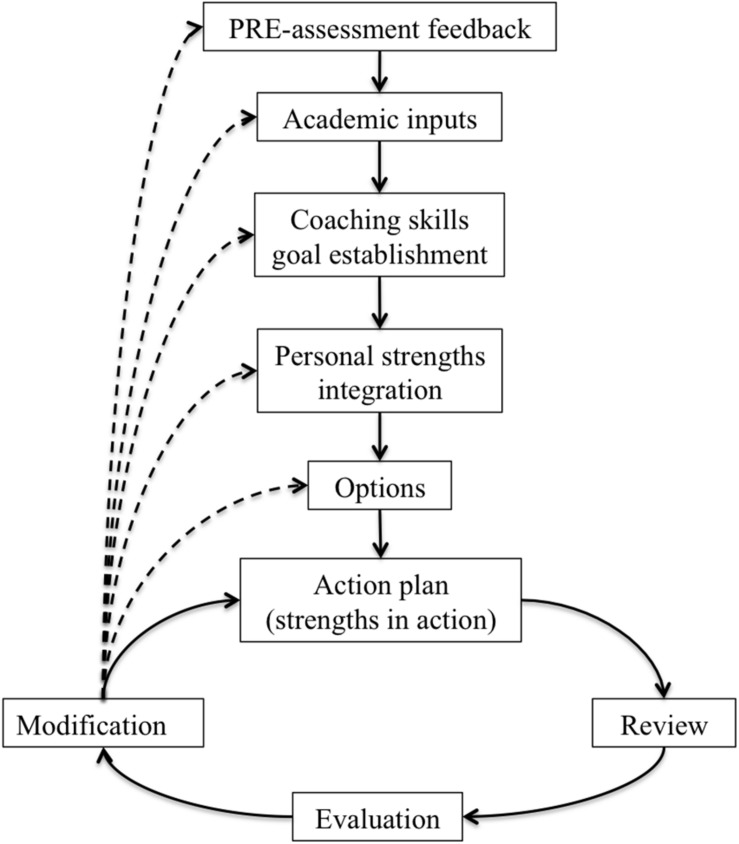
Coaching-based leadership intervention program model.

Based on the coaching literature (Spence and Grant, 2007), the strengths-based coaching approach (Govindji and Linley, 2007) and the Review, Evaluate, Goal, Reality, Option, Wrap-up (RE-GROW) model (see Whitmore, 1992; Grant, 2011 for further review), the coaching process was structured in seven phases: (1) feedback and insight into PRE-assessment results; (2) establishing specific goals related to the development or improvement of coaching-based leadership skills; (3) awareness and development of personal strengths; (4) identifying options in order to achieve the goal; (5) formulating an action plan based on the use of personal resources and strengths for goal achievement; (6) reviewing and evaluating progress: each coaching session started with a process of monitoring and evaluating the learning and actions completed since the last session; and (7) modifying action plans based on the previous evaluation. Finally, between sessions, specific exercises were used to practice the skill set they were developing at work. The Coaching-based Leadership Intervention Program model is summarized in Figure 2.

### Measures

#### Coaching-Based Leadership Skills

Based on the existing literature and research, a 12-item scale assessing eight essential coaching-based leadership skills classified into four dimensions was developed for the purpose of this particular study: (1) Working alliance, which consists of one skill (developing a working alliance) with two items based on the genuineness of the relationship subscale of the full Perceived Quality of the Employee Coaching Relationship scale (Gregory and Levy, 2010); (2) Open communication, which consists of two skills: active, empathic, and compassionate listening with three items based on the Compassionate Scale (Pommier, 2010) and powerful questioning with one item based on the communication dimension of the Coaching Skills Scale (Baron and Morin, 2009; (3) Learning and development, which consists of three skills: facilitate development and providing feedback with one item each based on the facilitate development subscale of the Managerial Coaching Skills Scale (Park et al., 2008), and strength spotting and development, with one item based on the ability and application subscales of The Strength Spotting Scale (Linley et al., 2010a); and (4) Progress and results, which consists of two skills: planning and goal setting and manage progress with one item each based on the Goal-Focused Coaching Skills Questionnaire (Grant and Cavanagh, 2007b). Sample items are listed in the Supplementary Appendix representing each dimension. Participants were asked to respond using a Likert scale ranging from 0 (*strongly disagree*) to 6 (*strongly agree*). The same measure was administrated to participants’ employees and supervisors, but in this case, respondents were asked to think about their perception of the participants’ skills. The scale was adapted and reworded, so that the referent was the leader who participated in the intervention (i.e., *“He/she is able to*…*”*).

The revised scale was next tested using confirmatory factor analysis via Mplus and reliability tests using SPSS. Confirmatory factor analysis was constrained to a four-factor model and resulted in an acceptable fit to the data in almost all indicators (self-reported scores: χ^2^ = 86.252; d.f. = 48; *p* = 0.00; TLI = 0.87; CFI = 0.90; RMSEA = 0.08; WRMR = 0.813; supervisors’ scores: χ^2^ = 88.702; d.f. = 48; *p* = 0.00; TLI = 0.97; CFI = 0.98; RMSEA = 0.09; WRMR = 0.734; employees’ scores: χ^2^ = 104.150; d.f. = 48; *p* = 0.00; TLI = 0.99; CFI = 0.99; RMSEA = 0.08; WRMR = 0.538). Additionally, the coefficient alpha for the whole scale showed high levels of internal consistency: 0.85 for self-reported scores, 0.94 for supervisors’ scores, and 0.97 for employees’ scores. The values for each dimension analyzed separately also indicated acceptable consistency: developing a working alliance (self-reported scores = 0.64; supervisors’ scores = 0.91; employees’ scores = 0.91); open communication (self-reported scores = 0.79; supervisors’ scores = 0.83; employees’ scores = 0.93); facilitating learning and development (self-reported scores = 0.79; supervisors’ scores = 0.86; employees’ scores = 0.93); manage progress and results (self-reported scores = 0.81; supervisors’ scores = 0.93; employees’ scores = 0.93).

#### Work Engagement

This variable was measured using the 9-item short version of the Utrecht Work Engagement Scale (Schaufeli et al., 2006). The scale consists of three dimensions (vigor, dedication, and absorption) with three items each (i.e., *“At my work, I feel bursting with energy”;* vigor). All the items were rated on a Likert scale ranging from 0 (*almost never*) to 6 (*almost always*).

#### Psychological Capital

This construct was assessed by the Psychological Capital Questionnaire (PCQ-12; Avey et al., 2011), adapted from the PCQ-24 scale (Luthans et al., 2007). The scale consists of 12 items with four dimensions (self-efficacy, measured with three items; hope, measured with four items; resilience, measured with three items; and optimism, assessed by two items (i.e., *“I look on the bright side of things regarding this situation”; optimism).* Participants were asked to rate each of the statements using a Likert scale ranging from 0 (strongly disagree) to 5 (strongly agree).

#### In- and Extra-Role Performance

This variable was assessed by six items included in the HERO (HEalthy and Resilient Organizations) questionnaire (Salanova et al., 2012), adapted from Goodman and Svyantek’s (1999) scale. Two different dimensions (in-role performance and extra-role performance) were considered, with three items in each (i.e., “*He/she helps other employees with their work when they have been absent”;* extra-role performance). Participants’ supervisors and employees were asked to rate each of the statements individually using a Likert scale ranging from 0 (strongly disagree/never) to 6 (strongly agree/always).

#### Qualitative Measure

In order to obtain data about their personal experiences with the program, participants were asked to respond to the following question during the last coaching session: *“What specific positive outcomes (if any) did you gain from participating in this program?”* The use of an open-question methodology is an important point in this study because it allows the participants to determine which issues they consider most beneficial (Grant and Hartley, 2014).

### Data Analyses

Different data analyses were conducted. First, internal consistencies (Cronbach’s alpha), descriptive analysis, and inter-correlations among the study variables were calculated. Then, one-factor Analyses of Variance (ANOVA) were performed, using SPSS, to discover whether there were significant differences between the executives and middle managers within the EX at the three evaluation times (PRE, POST, and FUP). Next, the same analyses were applied to examine whether there were significant differences in the study variables between the EX and WL prior to the intervention.

In order to test the effects of the intervention program, data were analyzed using 2 × 2 repeated-measures ANOVA, consisting of one between-subjects factor (group: EX, WL) and one within-subjects factor (time: Time 1; T1, and Time 2; T2). In this comparison, T1 refers to the first pre-intervention assessment for both EX and WL, whereas T2 refers to the post-intervention assessment for EX and the second pre-intervention assessment for WL, just before this group starts the program. The FUP time factor could not be considered when comparing the two groups. For organizational reasons, the WL had completed the intervention before the EX filled out the FUP assessment.

For supervisors’ data, the same analyses were performed as in the self-reported data. However, for the employees’ data, because responses were not identifiable, 2 × 2 repeated-measures could not be performed, and so univariate analysis was applied to employees’ scores to examine interaction effects by comparing the whole means between T1-T2 for each group (EX and WL) separately.

Moreover, once the WL group had completed the intervention program, paired-sample *t*-tests were carried out for the whole intervention group (EX and WL; *N* = 37) to test for differences between PRE, POST, and FUP time factors. In this comparison, T1 referred to the PRE assessment for the EX, whereas T2 referred to the PRE assessment for the WL, that is, the evaluation applied just before this latter group started the intervention. For these analyses, both self-reported and supervisors’ scores were used. Next, to test for differences in employees’ scores across the three time factors, univariate analyses were performed.

Following Cohen (1988), eta squared in the repeated-measures ANOVA and Cohen’s *d* as a measure of effect sizes (small effect = 0.1–0.3; moderate or intermediate effect = 0.3–0.5; large effect = > 0.5) in paired-sample *t*-tests were estimated, in addition to *t*-test comparisons between groups.

Finally, qualitative data on the outcomes of the intervention program were analyzed using the interpretive content analysis, proposed for coding texts into categories and counting the frequencies in each category (Ahuvia, 2001). This method is used to analyze categories and obtain conclusions based on a previous theoretical framework (Denecke and Nejdl, 2009). First, each leader’s response was carefully analyzed and incorporated into a database. Next, responses were systematically classified and grouped according to thematic content. At this stage, a construction of themes emerged for the whole group of participants. Finally, the frequency of each emerging theme was estimated.

## Results

Means, standard deviations, internal consistencies (Cronbach’s alpha), and correlations among the study variables for PRE, POST and FUP intervention scores are shown in Table 2 for self-reported scores, Table 3 for supervisors’ scores, and Table 4 for employees’ scores. Next, one-factor ANOVA results showed that there were no significant differences in self-reported variables between the executives and middle managers in the EX at the PRE intervention time [coaching-based leadership skills: *F*(1,24) = 0.31; *p* = 0.58, *ns*; PsyCap: *F*(1,24) = 1.92; *p* = 0.18, *ns;* work engagement: *F*(1,24) = 0.17; *p* = 0.68, *ns*]. Moreover, one-factor ANOVA results comparing the EX and WL revealed no significant differences between the two groups on the same variables at PRE intervention [coaching-based leadership skills: *F*(1,40) = 0.24; *p* = 0.88, *ns*; PsyCap: *F*(1,40) = 0.41; *p* = 0.53, *ns;* work engagement: *F*(1,40) = 0.86; *p* = 0.36]. With these results, we proceeded to carry out the study with both groups included in the same sample.

**TABLE 2:** PRE, POST, and FUP self-reported means, standard deviations, internal consistencies, and correlations of all variables for the whole intervention group.

Variables	M	SD	α	1	2	3	4	5	6	7	8	9
**PRE intervention scores**												
1. Coaching-based leadership skills	4.80	0.48	0.85	–								
2. PsyCap	4.15	0.44	0.82	0.57**	–							
3. Work engagement	4.85	0.71	0.86	0.52**	0.56**	–						
**POST intervention scores**												
4. Coaching-based leadership skills	4.92	0.41	0.84	0.68**	0.43**	0.26*	–					
5. PsyCap	4.40	0.33	0.79	0.21*	0.45**	0.27*	0.35*	–				
6. Work engagement	5.12	0.55	0.93	0.13	0.33**	0.73**	0.31*	0.43**	–			
**FUP intervention scores**												
7. Coaching-based leadership skills	4.97	0.53	0.92	0.67**	0.54**	0.33*	0.58**	0.38*	0.12	–		
8. PsyCap	4.27	0.47	0.87	0.28*	0.38**	0.22*	0.15	0.52**	0.27	0.56**	–	
9. Work engagement	4.96	0.74	0.90	0.11	0.20*	0.38*	0.13	0.23*	0.53**	0.24*	0.45**	–

Correlations; **p < 0.01; *p < 0.05.

**TABLE 3:** PRE, POST, and FUP supervisor score means, standard deviations, internal consistencies, and correlations of all variables for the whole intervention group.

Variables	M	SD	α	1	2	3	4	5	6	7	8	9
**PRE intervention scores**												
1. Coaching-based leadership skills	4.21	0.90	0.94	–								
2. In-role performance	4.69	0.96	0.94	0.66**	–							
3. Extra-role performance	5.00	0.96	0.90	0.62**	0.71**	–						
**POST intervention scores**												
4. Coaching-based leadership skills	4.51	0.84	0.93	0.88**	0.58**	0.53**	–					
5. In-role performance	4.90	0.75	0.87	0.65**	0.82**	0.67**	0.65**	–				
6. Extra-role performance	5.22	0.69	0.83	0.39*	0.38*	0.66**	0.49**	0.55**	–			
**FUP intervention scores**												
7. Coaching-based leadership skills	4.6	0.86	0.94	0.78**	0.72**	0.46**	0.79**	0.65**	0.33*	–		
8. In-role performance	5.00	0.94	0.93	0.39*	0.72**	0.51**	0.45**	0.61**	0.34*	0.73**	–	
9. Extra-role performance	5.14	0.72	0.81	0.48**	0.56**	0.67**	0.58**	0.66**	0.77**	0.55**	0.61**	–

Correlations; **p < 0.01; *p < 0.05.

**TABLE 4:** PRE, POST, and FUP employee score means, standard deviations, internal consistencies, and correlations of all variables for the whole intervention group.

Variables	M	SD	α	1	2	3
**PRE intervention scores**						
1. Coaching-based leadership skills	4.19	1.38	0.97	–		
2. In-role performance	4.55	1.26	0.94	0.84**	–	
3. Extra-role performance	4.32	1.35	0.87	0.82**	0.83**	–
**POST intervention scores**						
1. Coaching-based leadership skills	4.76	0.95	0.96	–		
2. In-role performance	4.94	1.03	0.94	0.79**	–	
3. Extra-role performance	4.82	1.03	0.87	0.76*	0.81**	–
**FUP intervention scores**						
1. Coaching-based leadership skills	4.98	0.66	0.92	–		
2. In-role performance	5.23	0.81	0.86	0.66**	–	
3. Extra-role performance	5.14	0.76	0.79	0.54**	0.79**	–

Correlations; **p < 0.01; *p < 0.05.

### Coaching-Based Leadership Skills

A repeated-measures ANOVA for coaching-based leadership skills showed no significant time (T1, T2) × group (EX, WL) interaction effects [*F*(1,38) = 2.11; *p* = 0.15, *ns*] for self-reported scores, although the levels were higher at T2 than at T1. Paired sample *t*-tests results for EX separately indicated no significant differences from T1 to T2 [*t*(23) = −1.883; *ns*] for self-reported scores. However, results showed significant differences from T1 to T4 (FUP) for this variable [*t*(22) = −2.604, *p* < 0.05, *d* = 1.11)], demonstrating a large effect size. Moreover, paired sample *t*-test results for WL indicated no significant differences from T1 to T2 [*t*(15) = −0.330; *ns*], as expected.

Results for supervisors’ scores indicated a significant time (T1, T2) × group (EX, WL) interaction effect [*F*(1, 33) = 17.78, *p* < 0.001, η_p_^2^ = 0.054], indicating statistically higher levels at T2 compared to T1. This result had an intermediate effect size. Paired sample *t*-tests results for EX separately indicated significant differences from T1 to T2 [*t*(19) = −5.233, *p* < 0.001, *d* = 2.40)] and from T1 to T4 (FUP) [*t*(18) = −5.316, *p* < 0.001, *d* = 2.50)], demonstrating large effect sizes. Whereas paired sample *t*-test results for WL indicated no significant differences from T1 to T2 [*t*(14) = −0.636; *ns*], as expected.

**FIGURE 3:**
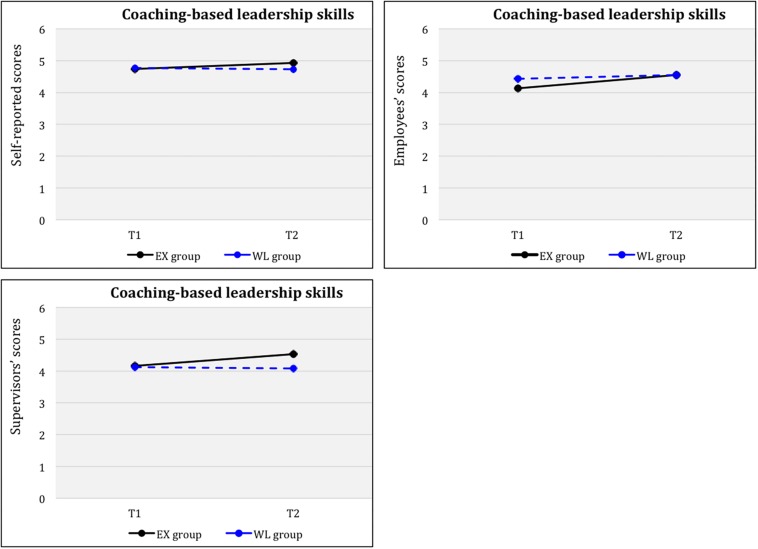
Coaching-based leadership skills for groups (EX, WL) across time (Tl, T2).

Additionally, univariate analysis of this variable was performed on employees’ scores to compare time factors for each group separately. Results showed that the EX group had significantly higher scores at T2 compared to T1 [*t*(195) = −2.31, *p* < 0.05, *d* = 0.33], with a intermediate effect size, whereas the WL group did not differ significantly from T1 to T2 [*t*(113) = −0.49; *ns*], as expected. Figure 3 shows plotted means for each time factor (T1, T2) across the groups (EX, WL) for self-reported, supervisors’, and employees’ scores.

Finally, paired-sample *t*-test results for the whole intervention group (*N* = 41) after the WL had completed the program indicated significant differences in the self-reported coaching-based leadership skills variable from PRE to POST [*t*(37) = −2.07, *p* < 0.05, *d* = 0.68] and from PRE to FUP [*t*(37) = −2.07, *p* < 0.05, *d* = 0.70]. In both cases, levels were significantly higher at the endpoint compared to baseline, and the effect sizes reported were moderate. In the case of supervisors’ scores, results also showed statistically significant higher levels at POST compared to PRE [*t*(34) = −4.08, *p* < 0.001, *d* = 1.39], and at FUP compared to PRE [*t*(32) = −3.51 *p* < 0.001, *d* = 1.24], with large effect sizes. Additionally, results from univariate analyses of employees’ scores indicated that the whole intervention group had significantly higher scores at POST [*t*(276) = −3.75, *p* < 0.001, *d* = 0.45] and FUP [*t*(252) = −4.93, *p* < 0.001, *d* = 0.62], compared to PRE, with intermediate effect sizes.

### PsyCap

**FIGURE 4:**
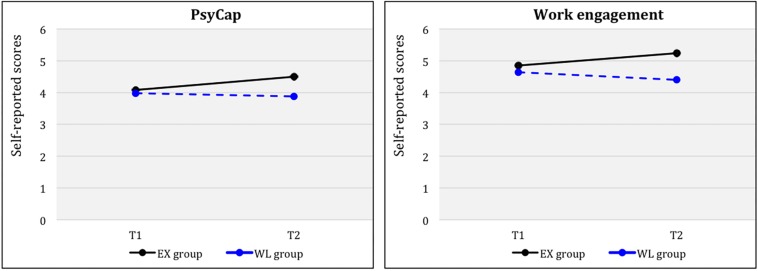
PsyCap and work engagement for groups (EX, WL) across time (Tl, T2).

A repeated-measures ANOVA of PsyCap showed a significant time (T1, T2) x group (EX, WL) interaction effect for self-reported scores [*F*(1, 38) = 6.78 *p* < 0.05, η_p_^2^ = 0.15], with a large effect size. Results indicated that the EX had statistically significant higher PsyCap scores than the WL at T2. Figure 4 shows plotted means for each time factor (T1, T2) across the groups (EX, WL) for self-reported scores. Paired sample *t*-tests results for EX separately indicated significant differences from T1 to T2 [*t*(23) = −3.699, *p* < 0.001, *d* = 1.54)] and from T1 to T4 (FUP) [*t*(22) = −2.798, *p* < 0.001, *d* = 1.19)], demonstrating large effect sizes. Additionally, paired sample *t*-test results for WL indicated no significant differences from T1 to T2 [*t*(15) = 0.629; *ns*], as expected.

Furthermore, paired-sample *t*-test results for the whole intervention group (*N* = 41) after the WL had completed the program indicated significantly higher self-reported scores for PsyCap at POST compared to PRE [*t*(37) = −3.65 *p* < 0.001, *d* = 1.20], with a large effect size. However, results showed no significant differences between PRE and FUP [*t*(34) = −0.94 *p* = 0.35; *ns*], although the levels were higher at FUP.

### Work Engagement

A repeated-measures ANOVA of work engagement showed a significant time (T1, T2) x group (EX, WL) interaction effect for self-reported scores [*F*(1, 38) = 10.9, *p* < 0.005, η_p_^2^ = 0.19], with a large effect size. Results indicated that the EX had statistically significant higher work engagement scores than the WL at T2. Figure 4 shows plotted means for each time factor (T1, T2) across the groups (EX, WL) for self-reported scores. Moreover, paired sample *t*-tests results for EX separately indicated significant differences from T1 to T2 [*t*(23) = −3.759, *p* < 0.05, *d* = 1.56)], demonstrating a large effect size. However, results showed no significant differences from T1 to T4 (FUP) for this variable [*t*(23) = −1.024; *ns*]. Additionally, paired sample *t*-test results for WL indicated no significant differences from T1 to T2 [*t*(15) = 1.374; *ns*], as expected.

Finally, paired-sample *t*-test results for the whole intervention group (*N* = 41) after the WL had completed the program indicated significantly higher self-reported scores for work engagement at POST compared to PRE [*t*(37) = −3.42 *p* < 0.05, *d* = 1.12], with a large effect size. However, results showed no significant differences between PRE and FUP [*t*(37) = −0.54; *ns*], although the levels were higher at FUP.

### In-Role and Extra-Role Performance

A repeated-measures ANOVA for performance showed no significant time (T1, T2) x group (EX, WL) interaction effects for supervisors’ scores [in-role performance: *F*(1, 33) = 1.88; *p* = 0.17, *ns*; extra-role performance: *F*(1, 33) = 1.7; *p* = 0.2, *ns*], although the levels were higher at T2 compared with T1. Moreover, paired sample *t*-tests results for EX separately indicated no significant differences from T1 to T2 [*t*(19) = −1.831; *ns*)], and significant differences from T1 to T4 (FUP) [*t*(18) = −2.394, *p* < 0.01, *d* = 1.13)], demonstrating a large effect size, for in-role performance. Additionally, results for extra-role performance for this group indicated significant differences from T1 to T2 [*t*(19) = −1.945, *p* < 0.05, *d* = 0.89)] and from T1 to T4 (FUP) [*t*(18) = −1.932, *p* < 0.05, *d* = 0.91)] demonstrating large effect sizes. Whereas paired sample *t*-test results for WL indicated no significant differences from T1 to T2 [in-role performance: *t*(14) = −0.626; *ns*; extra-role performance: *t*(14) = 0.118; *ns*], as expected.

**FIGURE 5:**
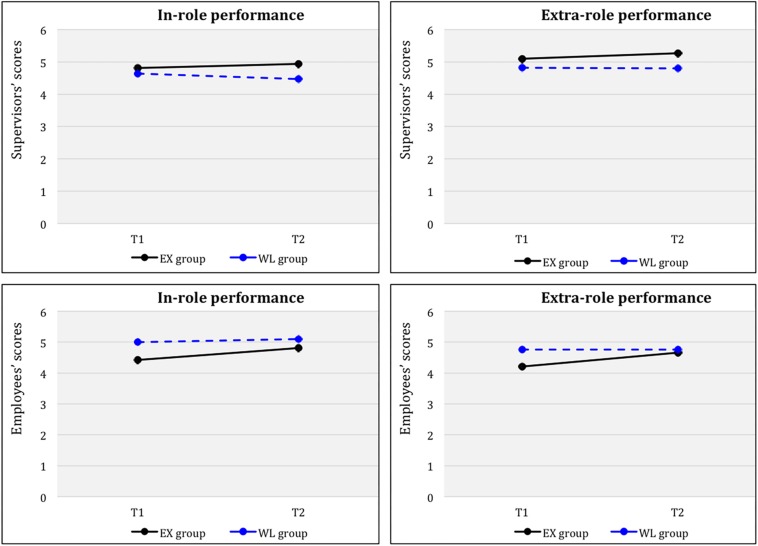
In-role and extra-role performance for groups (EX, WL) across time (Tl, T2).

Additionally, univariate analysis of this variable was performed on employees’ scores to compare the time factors for each group separately. Results showed that the EX had significantly higher scores at T2 [in-role performance: *t*(195) = −2.24, *p* < 0.05, *d* = 0.32; extra-role performance: *t*(195) = −2.24, *p* < 0.05, *d* = 0.32] compared to T1 (with an intermediate effect size), whereas the WL did not differ significantly from T1 to T2 [in-role performance: *t*(90) = −0.69; *ns*; extra-role performance: *t*(90) = 0.005; *ns*]. Figure 5 shows plotted means for each time factor (T1, T2) across the groups (EX, WL) for supervisors’ and employees’ scores.

Finally, paired-sample *t*-tests were carried out for the whole intervention group (*N* = 41) after the WL had completed the program. Results for supervisors’ scores showed significantly higher levels at POST compared to PRE [in-role performance: *t*(33) = −2.20 *p* < 0.05, *d* = 0.77; extra-role performance: *t*(33) = −1.98 *p* < 0.05, *d* = 0.69], with intermediate effect sizes; and at FUP compared to PRE [in-role performance: *t*(30) = −2.48 *p* < 0.05, *d* = 0.90; extra-role performance: *t*(30) = −1.84 *p* < 0.05, *d* = 0.67], with large and intermediate effect sizes, respectively. Additionally, results of univariate analyses of employees’ scores indicated that the whole intervention group had significantly higher scores at POST compared to PRE [in-role performance: *t*(277) = −2.65, *p* < 0.05, *d* = 0.32; extra-role performance: *t*(277) = −3.22, *p* < 0.001, *d* = 0.39], with intermediate effect sizes; and at FUP compared to PRE [in-role performance: *t*(253) = −4.54, *p* < 0.001, *d* = 0.57; extra-role performance: *t*(253) = −5.18, *p* < 0.001, *d* = 0.65], with moderate effect sizes. Moreover, results also showed significantly higher scores at FUP compared to POST [in-role performance: *t*(196) = −2.20, *p* < 0.05, *d* = 0.31; extra-role performance: *t*(196) = −2.46, *p* < 0.05, *d* = 0.35], with an intermediate effect size.

**FIGURE 6:**
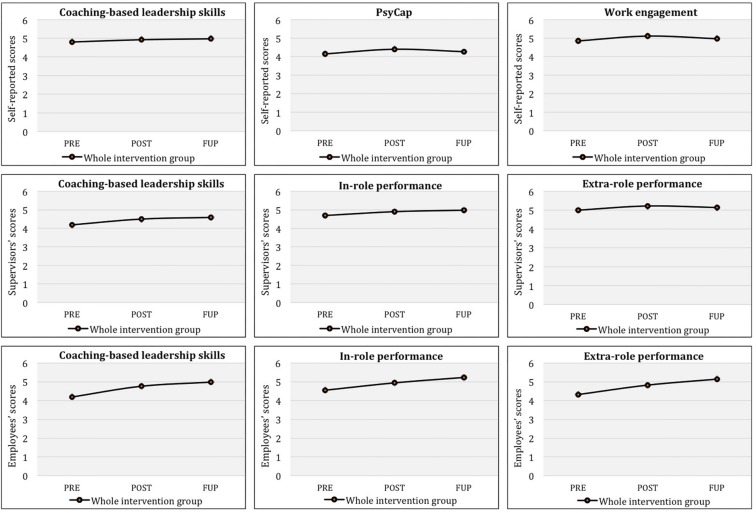
Coaching-based leadership skills, PsyCap, work engagement, in-role and extra-role performance for the whole intervention group across time.

Figure 6 shows the study variables’ plotted means for the whole intervention group (*N* = 41) for self-reported, supervisors’, and employees’ scores. Means and standard deviations for each variable across both groups at different times (T1 and T2) are shown in Table 5.

**TABLE 5:** T1 and T2 means and standard deviations (SD) for the EX and the WL.

	EX (*N* = 23)				WL (*N* = 15)			
	T1	T2	*t-*value	*p-*value	T1	T2	*t-*value	*p-*value
**Self-reported scores**								
Coaching-based leadership skills	4.7 (0.50)	4.9 (0.37)	−1.89	0.072	4.7 (0.46)	4.7 (0.51)	0.33	0.746
PsyCap	4.0 (0.50)	4.5 (0.34)	−3.69	0.001	4.0 (0.53)	3.9 (0.73)	0.63	0.54
Work engagement	4.8 (0.68)	5.2 (0.51)	−3.76	0.001	4.6 (0.78)	4.4 (1.25)	1.37	0.190
**Supervisors’ scores**								
Coaching-based leadership skills	4.1 (0.81)	4.5 (0.82)	−5.23	0.000	4.1 (1.02)	4.1 (0.98)	0.63	0.535
In-role performance	4.8 (0.77)	4.9 (0.73)	−1.83	0.083	4.6 (1.05)	4.5 (1.13)	0.63	0.540
Extra-role performance	5.1 (0.83)	5.3 (0.66)	−1.94	0.067	4.8 (0.80)	4.8 (1.12)	0.12	0.908
**Employees’ scores**								
Coaching-based leadership skills	4.1 (1.38)	4.6 (1.05)	−2.31	0.022	4.3 (1.28)	4.5 (1.34)	–0.49	0.620
In-role performance	4.4 (1.29)	4.8 (0.99)	−2.24	0.026	4.9 (1.00)	5.1 (0.82)	–0.70	0.483
Extra-role performance	4.2 (1.37)	4.6 (1.17)	−2.35	0.019	4.7 (1.20)	4.7 (1.15)	0.05	0.996

### Qualitative Data

All the participants (*N* = 37) answered a qualitative question (*“What specific positive outcomes (if any) did you gain from participating in this program?”*) during the last individual coaching session. The following themes emerged and are listed below according to the frequency with which they were mentioned by the participants (note: some participants gave more than one response): (1) Awareness and professional insight (28 responses: 23.8%; e.g., *“Awareness of how I see myself as a leader and how others see me”*); (2) Development/increases in coaching-based leadership skills (17 responses: 14.4%; e.g., *“Greater capacity to listen and ask employees powerful questions”*); (3) Increased self and/or team performance (16 responses: 13.6%; e.g., *“The program has followed the plant’s continuous improvement line, such as IDP; Indicators for Personal Development”*); (4) Increased personal strengths/resources (14 responses: 11.9%; e.g., *“Being aware of how employees see me in the role of leader has increased my humility and open-mindedness”*); and (5) Positive changes in the environment (10 responses: 8.5%; e.g., *“I am getting more signs of optimism from co-workers, and with better predisposition to help others”*).

## Discussion

This study examined the impact of participating in a Coaching-based Leadership Intervention Program on coaching-based leadership skills, PsyCap, work engagement, and in-role and extra-role performance. Overall, the results of the study revealed that the intervention program is a successful strategy for improving the participants’ outcome variables (self-reported and assessed by their employees and supervisors) after participating in the program and 4 months after finishing it. In other words, managers and middle managers that trained to develop a coaching-based leadership style, improved their coaching-based leadership skills (i.e., develop a working alliance, active, empathic, and compassionate listening, powerful questioning, facilitate development, provide feedback, strengths spotting and development, support in planning and goal setting, and manage progress), and increased their levels of positive PsyCap (i.e., self-efficacy, hope, resilience, and optimism), work engagement (vigor, dedication, and absorption), and in-role and extra-role performance.

This study makes several contributions to the coaching-based leadership development literature. First, this is the first empirical study to evaluate and confirm the positive effects of a coaching-based leadership intervention on increasing the levels of the leaders’ coaching skills, PsyCap, work engagement, and in-role and extra-role performance. Since the coaching-based leadership term remains undertheorized (Berg and Karlsen, 2016), and its value and meaning within the organizational context have not been sufficiently captured (Dahling et al., 2016), findings of the current study can notable contribute to research on the benefits of this relatively new style of leadership. Additionally, identifying the attributes and outcomes that are most frequently associated with coaching-based leadership may allow for insight into the concept and further theory development (Cox et al., 2010).

Second, considering that previous research has focused on the impact of leadership development interventions on employees’ variables (Grant, 2010), in this study we focused on the leaders’ levels of the study variables (in a 360-degree assessment). Of the few studies that have examined the impact of a coaching leadership (Moen and Skaalvik, 2009; Grant and Hartley, 2014) or managerial coaching intervention (David and Matu, 2013; Ratiu et al., 2017) on the leaders’ own performance, none of them considered task and contextual performance separately. An additional contribution of this study is the innovative approach implemented during the intervention program aim to support the development and improvement of the managers’ coaching-based leadership skills. To achieve this goal and enhance positive outcomes, we followed a combination of workshop format, strengths-based leadership coaching, and practicing the skillset on-the-spot.

Fourth, this study extends the limited existing literature on empirical controlled trials with a 360-degree format using mixed methodologies to examine the efficacy of these intervention programs over time (longitudinal study; Grant, 2010). Given the importance of understanding the perceived benefits of participating in a leadership intervention and adopting coaching-based leadership skills in the workplace (Grant, 2010; Milner et al., 2018), a strength of this study is the exploration of the perceived outcomes of participating in the intervention using a qualitative methodology. Previous researchers have highlighted the potential usefulness of mixed methods for achieving a broader high-quality evaluation of interventions and providing a better understanding of research (Abildgaard et al., 2016). Lastly, considering the current lack of effectiveness (Lacerenza et al., 2017) and success in applying coaching-based leadership skills back in the workplace (Moen and Federici, 2012), in the current study we also analyzed the durability of the effects over time.

### Post-intervention Effects

Results for coaching-based leadership skills partially supported H1 of the study. Findings indicated statistically significant higher supervisor scores after finishing the intervention, comparing the two groups (experimental and waiting-list control), and for the whole intervention group. Employee scores showed that, although there were no significant differences between the two groups at T1 and T2, the experimental group significantly increased their coaching-based leadership skills after the intervention program compared to their baseline levels. Additionally, employees’ scores for the whole intervention group also increased significantly after finishing the intervention. Moreover, participants’ self-reported levels for the whole intervention group increased significantly after finishing the program. However, self-reported increased levels of this variable were not statistically significant after finishing the program the experimental group compared to waiting-list. This result may be explained by the insight participants gained after receiving feedback from the pre-assessment about how they are seen by their employees. Additionally, this result is in line with prior research, which emphasized that leaders need at least 3 months to assimilate and feel really comfortable with using coaching skills in the workplace (Grant and Hartley, 2013). In line with this statement, we understand that, first, there might have been a process of self-discovery and consciousness-raising, followed by long-term assimilation of the coaching skills and application in their daily work. However, it is worth mentioning that results for the whole intervention group demonstrated a positive impact with significant differences in self-reported coaching-based leadership skills after finishing and 4 months after finishing the intervention compared to the baseline levels. Furthermore, the use of supervisor and employee ratings, which indicated a significant increase in leaders’ coaching skills, help to support H1.

Overall, self-reported, employees’, and supervisors’ scores significantly increased after finishing the program in the whole intervention group, which helped to confirm H1. Additionally, participants’ qualitative responses also supported H1 for one of the expected outcomes of the program (i.e., “*development and increases in coaching-based leadership skills*”). Participants reported a greater capacity to enhance the strengths of their employees, help them achieve goals, and make them grow. Some of them also reported more authenticity in their role as coach, greater closeness in the relationship, and an increased ability to communicate by using effective listening and questioning techniques. Both the quantitative and qualitative results suggest the importance of helping leaders to develop and increase coaching skills (i.e., developing a working alliance and trust environment, open communication, facilitating learning and development, managing progress and results) in the workplace. The results on the impact of the implemented intervention on coaching skills are aligned with past research specifying the effectiveness of these development programs for leaders (Ellinger et al., 2010, 2011; Grant, 2010; David and Matu, 2013; Cummings et al., 2014; Grant and Hartley, 2014). Overall, the Coaching-based Leadership Intervention Program can be recommended for implementation in organizational settings due to the set of tools it provides and its effective methodology for enhancing coaching skills that interact in the workplace.

Regarding the effects of the intervention on PsyCap and work engagement, the results fully supported H2 and H3, respectively; that is, participants’ self-reported levels of PsyCap and work engagement increased significantly after participating in the program, both compared to the WL (from T1 to T2) and considering the whole intervention group (from PRE to POST). These findings suggest that training in core coaching skills, such as developing a warm and trusting environment among employees, generating effective communication, delivering meaningful and positive feedback, and helping them to discover and use strengths and achieve valuable goals and action plans, leads leaders to develop their personal resources (i.e., PsyCap), and increase their levels of energy, absorption, and dedication to the job. This is important because a resourceful work environment (i.e., coaching provided by the leader and opportunities for professional development) stimulate personal growth through the development of self-efficacy, hope, resilience, and optimism, which in turn lead to higher work engagement (Luthans et al., 2006; Xanthopoulou et al., 2007). Additionally, employees with high levels of engagement are likely to make more effort in their tasks and be more efficient (Kark, 2011; Llorens-Gumbau and Salanova-Soria, 2014).

Findings for the impact of the intervention program on PsyCap are consistent with previous research that found a positive direct relationship between job resources (i.e., coaching provided by the leader and opportunities for professional development) and personal resources (i.e., self-efficacy, organizational-based self-esteem and optimism; Xanthopoulou et al., 2007), and between managerial coaching and employees’ PsyCap (Hsu et al., 2019). However, there are still no studies that examined or coaching leaders and their own levels of PsyCap in cross-sectional and quasi-experimental studies. Thus, the present study represents a step forward with respect to previous research in analyzing and confirming the effect of leaders developing a coaching-based leadership style on their levels of PsyCap after participating in a training intervention. Moreover, our findings for the impact of the intervention on work engagement are in line with previous research that found a positive link between this variable and the leader’s coaching (Lin et al., 2016; Ladyshewsky and Taplin, 2017, 2018; Ali et al., 2018; Lee et al., 2018; Tanskanen et al., 2019). Despite the increasing number of studies exploring this link, work engagement has mostly been evaluated in non-experimental cross-sectional studies and as an employee-related outcome. Thus, our study provides an innovate approach by evaluating the effect of the intervention on the leaders’ work engagement. Additionally, participants’ qualitative responses helped to support H2 and H3 about two of the expected outcomes of the intervention (i.e., “*increased personal strengths/resources”* and *“positive changes in the environment”*). Specifically, the responses revealed that the program was a valuable tool in helping individuals to gain awareness and insight into personal resources and strengths, and produce positive changes in the work environment (i.e., quality of life, well-being, optimism, better communication).

Furthermore, the results for performance partially supported H4a and H4b. Particularly, supervisors’ perception of participants’ in-role and extra-role performance was higher for the experimental group after finishing the program, compared to the waiting-list control group, although the differences were not significant. However, employees’ perception of both in- and extra-role performance was significantly higher after finishing the intervention, compared to the waiting-list control group. These results may be explained by the fluent interaction during the intervention between the participants and their employees while applying the coaching skills at work. Therefore, employees observed a short-term improvement in their leaders’ performance after finishing the intervention, compared to the supervisors’ assessment, which may have required more time to perceive any significant change in the leaders’ performance. This last interpretation is confirmed by H5d. Precisely, supervisors perceived a significant increase in the participants’ in-role and extra-role performance levels 4 months after finishing the program. Additionally, both supervisors’ and employees’ scores for the whole intervention group were significantly higher after finishing the program.

Findings for the impact of the intervention program on in-role and extra-role performance are consistent with previous research that found a positive link between leaders’ as coaches skills and task-related performance (Ellinger et al., 2003, 2005, 2011; Gray, 2006; Grant and Cavanagh, 2007a; Agarwal et al., 2009; Grant et al., 2009; Liu and Batt, 2010; Kim, 2014; Kim and Kuo, 2015) and employees’ contextual-related performance (Ellinger and Cseh, 2007; Kim and Kuo, 2015). However, there are still few empirical studies examining the impact of coaching-based leadership interventions on leaders’ in-role and extra-role performance, and so our study contributes to and extends this aspect to the coaching-based leadership literature. Additionally, participants’ qualitative responses helped to support H3 about one of the expected outcomes of the intervention (i.e., “*increased performance levels*”). Specifically, the intervention appears to be a valuable method for improving leaders’ productivity and their teams’ performance, as reported by the participants.

### The Durability of the Effects

Taking into account the durability of the effects (FUP) in the whole intervention group, the findings fully confirmed H5a; that is, self-reported, supervisors’, and employees’ scores given for coaching-based leadership skills significantly increased at FUP compared to PRE intervention time. These results are consistent with previous research confirming that leaders need at least 3 months to develop and feel comfortable with using coaching skills in the workplace (Grant and Hartley, 2013). However, H5b and H5c were not supported, indicating that although participants’ levels of work engagement and PsyCap were higher 4 months after finishing the intervention, compared to the baseline levels, the differences were not significant, and so the effects were not sustained for these two variables. Finally, the study findings fully supported H5d. Specifically, supervisors’ and employees’ perceptions of leaders’ in-role and extra-role performance levels increased significantly 4 months after finishing the program, compared to PRE intervention time. Additionally, employees also perceived a significant increase in participants’ performance at FUP compared to POST time. Although this was not included in our hypotheses, it is worth mentioning because it demonstrates a strong trend toward improvement in leaders’ performance over time, as perceived by their employees.

### Theoretical and Practical Implications

This study has a number of theoretical implications. First, it contributes to the coaching and leadership framework alliance by exploring its conceptualization, structure, and the processes inherent in its development (Kemp, 2009). The study presents a rigorous and consistent empirical design that examines behaviors and skills of this relatively new form of leadership in the work environment (Batson and Yoder, 2012). Second, findings offer empirical support for the potential benefits of a coaching-based leadership style in organizations, advancing the theoretical understanding of its positive influence on work-related outcomes (i.e., PsyCap, work engagement, and in-role and extra-role performance).

Third, results from the present study contribute to the JD-R model (Bakker and Demerouti, 2007), confirming both the intrinsic motivational role of coaching-based leadership as a job resource that enhances personal resources (i.e., PsyCap), and work engagement, and its extrinsic motivational role fostering task performance. Additionally, the study findings extend this model by demonstrating the potential role of coaching-based leaders in fostering extra-role performance. In sum, leaders who train in developing a coaching-based leadership style (job resource), tend to increase their levels of positive PsyCap (personal resource), that is they expect good thing to happen at work, believe they can perform effectively, are more confident in accepting challenging tasks, are motivated to work hard when they encounter difficulties, proactively plan for alternative pathways for task accomplishment, and are able to rebound and start over when needed (Youssef and Luthans, 2012). Additionally, the development of a coaching-based leadership style and personal resources stimulate a motivational process that leads to higher levels of energy, absorption, and dedication to the job, and higher task and contextual performance.

Fourth, the intervention presented in this study contributes to the positive psychology literature through the development of an effective intervention methodology based on a strengths-based coaching approach (Biswas-Diener and Dean, 2007; MacKie, 2014). It also extends this approach by pairing personal strengths with coaching-based leadership skills and aligning them with goal achievement. Finally, findings from this study also help to confirm that strengths-based coaching can be effective, even when the number of coaching sessions is relatively low (Theeboom et al., 2014; Peláez et al., 2019).

In terms of practical implications, given the little guidance that coaching leaders receive in their own growth and development (Kemp, 2009), this study addresses useful tools and techniques that can be used by practitioners or Human Resources professionals to teach and train the development of coaching-based leadership and, therefore, increase the effectiveness of leadership and work-related outcomes in organizations. Another practical implication is the potential for short-term coaching sessions to help improve coaching-based leadership skills, PsyCap, personal strengths, work engagement, well-being, and performance in work settings. In line with previous research that have indicated that 47% of line managers use coaching in their work, this study highlights the organizational need to build internal coaching capability in leaders (Hsu et al., 2019). This is important because as a result of the alliance-building process, both the leader and the employee collaborate to develop performance goals and new ways to achieve them (Kemp, 2009).

### Limitations and Directions for Future Research

Although interesting results were obtained, the present study also has some limitations. First, the groups were not randomly chosen for the experimental condition because the middle managers in the study were line managers for whom the executives had management responsibilities. Thus, the company decided to separate the two groups. However, one-factor ANOVA results showed that there were no significant differences in any of the variables between the executives and middle managers in the experimental group on the PRE, POST, and FUP assessments. Moreover, previous studies highlighted the need to reinforce the link between research and professional practice, while considering the company or organization’s characteristics, preferences, and requirements, in order to implement interventions (Tkachenko et al., 2017; Ortega-Maldonado, 2018).

Second, the sample size is not large enough to make assumptions about the general efficacy of the intervention. However, previous research stated that statistical significance can also be influenced by small sample sizes (Cumming, 2014). In line with this assumption, the majority of the effects obtained were significant, with moderate to large effect sizes, and the findings were novel. Moreover, this study aimed to be useful for both practitioners and researchers in terms of scientific accuracy, while approaching fieldwork activities as much as possible. Qualitative data were also obtained to reinforce and confirm the study conclusions. However, future research should extend and replicate this study in more diverse and larger samples to improve the generalizability of the results.

Third, due to an organizational decision, employees’ answers to the questionnaires were anonymous, and responses were not identifiable over time. Additionally, some of the participants were supervisors or employees of other participants. This unbalanced sample may lead to non-independence in the study measures and experimental assignments. However, in the assessment, both supervisors and employees were asked to assess the leaders’ skills and performance in their specific roles in the company, rather than the observed changes from the intervention.

A fifth limitation is that the research design had to be adapted to the organizational context and requirements, and so some adjustments were made. For instance, the waiting-list control group started the program immediately after the experimental group finished, and so comparisons of the two conditions at FUP could not be assessed. Although scores remained higher than baseline levels for the whole intervention group, the levels of some of the study outcomes (self-reported PsyCap and work engagement, and extra-role performance assessed by the supervisors) showed a decreasing pattern at FUP compared to POST-assessment. Therefore, future studies should include follow-up coaching sessions over time in order to maintain and optimize the outcome variables.

As a complementary approach, it would be interesting for future studies to include diary studies in order to obtain relevant information about the underlying psychological mechanisms throughout the program that can influence the outcome variables (i.e., PsyCap, work engagement). Future studies could also evaluate the impact of such programs on employees’ variables of well-being and performance, in addition to objective organizational performance metrics. Finally, future controlled-trial studies should conduct research comparing coaching-based leadership interventions with other interventions, such as self-development tools from positive psychology, and with control groups, in order to explore and compare the effects on work-related outcomes.

## Data Availability Statement

The datasets generated for this study are available on request to the corresponding author.

## Ethics Statement

The studies involving human participants were reviewed and approved by the University Research Ethics Committee Universitat Jaume I. The patients/participants provided their written informed consent to participate in this study.

## Author Contributions

All the authors listed have made a substantial intellectual contribution to the research. MP and MS conceived the idea for the study and developed the study design. MP coordinated the entire intervention process, performed the data collection, conducted the analyses, and wrote the manuscript. MS contributed during the intervention program. MS and IM contributed to the interpretation of the results and revised the manuscript.

## Conflict of Interest

The authors declare that the research was conducted in the absence of any commercial or financial relationships that could be construed as a potential conflict of interest.

## Abbreviations

ANOVA: analyses of variance
EX: experimental group
FUP: follow up time
JD-R: job demands-resources
POST: post-assessment time
PRE: pre-assessment time
PsyCap: psychological capital
WL: waiting-list control group.
