# Supplementary material for: Coaching-Based Leadership Intervention Program: A Controlled Trial Study
Source: Front Psychol. 2020 Jan 30;10:3066. doi: 10.3389/fpsyg.2019.03066 (PMC7011779; doi:10.3389/fpsyg.2019.03066)
Supplement: Supplementary file 1 [file Data_Sheet_1.docx]

# APPENDIX

Coaching-based Leadership Skills Scale sample items for leader’s version

| 1. I am able to develop a climate of mutual respect with my employees (developing a working alliance). |
| --- |
| 1. I pay close attention when my employees talk to me (active, empathic, and compassionate listening). 2. I ask questions that help employees to better understand their situation, identify causes, and see possible improvement actions (powerful questioning). 3. My employees’ learning and development is one of my main responsibilities (facilitate development). 4. I constantly provide feedback to employees in order to improve their performance (providing feedback). 5. I find it easy to identify employees’ strengths, and help them use and develop new strengths (strength spotting and development). 6. I am very good at helping employees establish goals and develop clear, simple, and achievable action plans (planning and goal setting). |
| 1. I adequately follow up and evaluate employees’ progress towards their goals (Manage progress). |
